# Shepherding DNA ends: Rif1 protects telomeres and chromosome breaks

**DOI:** 10.15698/mic2018.07.639

**Published:** 2018-05-17

**Authors:** Gabriele A. Fontana, Julia K. Reinert, Nicolas H. Thomä, Ulrich Rass

**Affiliations:** 1 Friedrich Miescher Institute for Biomedical Research, Maulbeerstrasse 66, CH-4058 Basel, Switzerland.; 2 University of Basel, Petersplatz 10, CH-4003 Basel, Switzerland.

**Keywords:** genome stability, telomere homeostasis, DNA double-strand break repair pathway choice, non-homologous end-joining, DNA replication timing

## Abstract

Cells have evolved conserved mechanisms to protect DNA ends, such as those at the termini of linear chromosomes, or those at DNA double-strand breaks (DSBs). In eukaryotes, DNA ends at chromosomal termini are packaged into proteinaceous structures called telomeres. Telomeres protect chromosome ends from erosion, inadvertent activation of the cellular DNA damage response (DDR), and telomere fusion. In contrast, cells must respond to damage-induced DNA ends at DSBs by harnessing the DDR to restore chromosome integrity, avoiding genome instability and disease. Intriguingly, Rif1 (Rap1-interacting factor 1) has been implicated in telomere homeostasis as well as DSB repair. The protein was first identified in *Saccharomyces cerevisiae* as being part of the proteinaceous telosome. In mammals, RIF1 is not associated with intact telomeres, but was found at chromosome breaks, where RIF1 has emerged as a key mediator of pathway choice between the two evolutionary conserved DSB repair pathways of non-homologous end-joining (NHEJ) and homologous recombination (HR). While this functional dichotomy has long been a puzzle, recent findings link yeast Rif1 not only to telomeres, but also to DSB repair, and mechanistic parallels likely exist. In this review, we will provide an overview of the actions of Rif1 at DNA ends and explore how exclusion of end-processing factors might be the underlying principle allowing Rif1 to fulfill diverse biological roles at telomeres and chromosome breaks.

## RIF1 STRUCTURE AND FUNCTION

**Figure 1:**
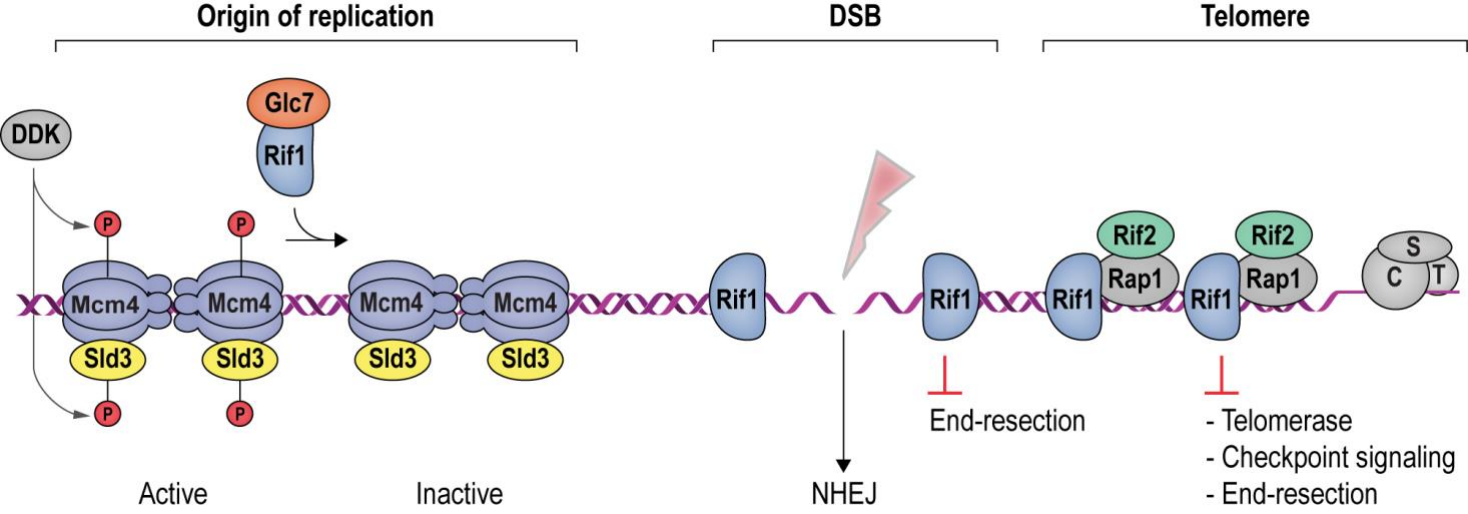
FIGURE 1: Rif1 is a multi-faceted genome maintenance factor. At origins of replication, Rif1 attenuates origin firing by recruiting PP1/Glc7, which reverses activating Mcm4 and Sld3 phosphorylation mediated by DDK (see **Box 1**). At DSBs, Rif1 tightly encases the break ends, gating access of the end-resection machinery. As a result, DSB ends are stabilized, promoting their re-ligation by NHEJ. At budding yeast telomeres, Rif1 forms part of an interlinked telosome protein meshwork with Rap1 and Rif2, underpinning telomere architecture and function. In addition, direct Rif1-DNA interactions are required to counteract telomerase and inadvertent checkpoint activation at chromosome ends (see text for details). CST denotes the Cdc13-Stn1-Ten1 complex bound to ssDNA telomeric overhangs.

**Figure 2:**
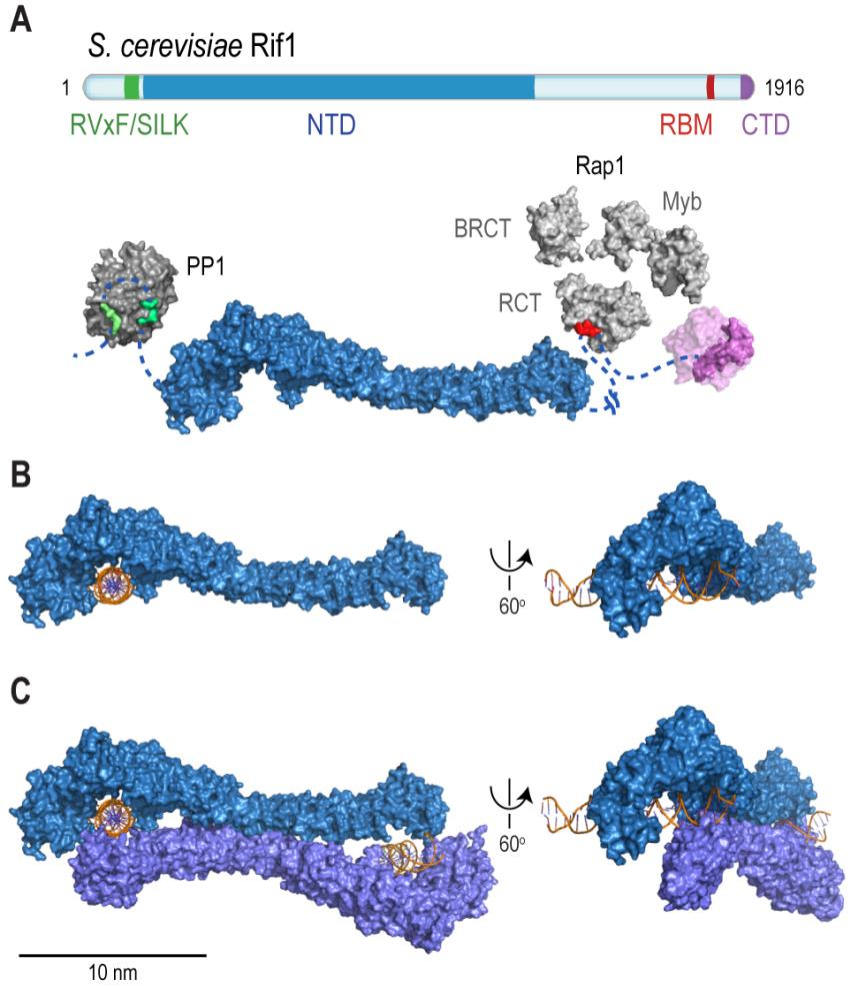
FIGURE 2: Rif1 domains and structural features. **(A)** Cartoon of *S. cerevisiae* Rif1 with structural representation of the indicated domains: RVxF/SILK PP1/Glc7 interacting motifs (*green*), NTD (N-terminal domain; *blue*), RBM (Rap1-binding motif; *red*) and CTD (C-terminal domain; *purple*). The RVxF (residues 115-118) and SILK (residues 146-149) motifs are shown bound with protein phosphatase PP1 (*dark grey*), modelled on two available co-crystal structures (PDB: 4G9J for RVxF, serine/threonine-protein phosphatase PP1-alpha catalytic subunit, *Homo sapiens* 32 and PDB: 2O8A for SILK, serine/threonine-protein phosphatase PP1-gamma catalytic subunit, *Rattus norvegicus *33). A flexible linker connects the RVxF and SILK motifs with the NTD (residues 188-1766), which is shaped like a shepherd’s crook (PDB: 5NVR 19). The NTD connects via a 462 residue unstructured linker with RBM (residues 1752-1772). A co-crystal structure of RBM with the Rap1 C-terminal domain (RCT) is depicted (PDB: 4BJT). In addition, Rap1 contains BRCT and Myb domains (represented in *light grey*, Rap1 linker regions between structured domains not shown). CTD (C-terminal domain of Rif1, residues 1857-1916, PDB: 4BJS 29) is a tetramerization domain, allowing oligomerization with other Rif1 molecules (as indicated in translucent* purple*). **(B)** The NTD of Rif1 in complex with dsDNA. **(C)** Rif1_NTD_ bound with two distinct DNA molecules in the head-to-tail dimer conformation observed in Rif1-DNA co-crystals and in solution. Contacts with the DNA are made by the concave surface of the so-called HOOK domain at the N-terminal end of the NTD (19(.

**Figure 3:**
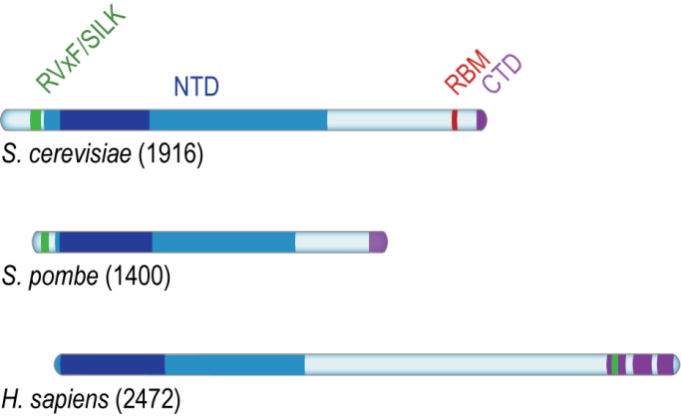
FIGURE 3: Conserved domains of Rif1. Rif1 orthologs from budding yeast (*S. cerevisiae*), fission yeast (*S. pombe*) and human (*H. sapiens*), aligned on the most conserved region of the protein (*dark blue*) present within the NTD, and corresponding to Pfam domain Rif1_N (residues 241-649 in *S. cerevisiae*, 108-471 in *S. pombe*, and 19-361 in *H. sapiens*). While the RVxF/SILK and NTD domains are found across organisms, RBM is only present in *Saccharomycetes*; the CTD domain is partially conserved from yeast to human (see text for details).

Rif1 is a multifaceted genome caretaker involved in telomere homeostasis, DSB repair pathway choice, and the regulation of replication timing (**Figure 1**). Rif1 orthologs in yeast 1,2 and higher eukaryotes 3,4,5,6,7 are divergent at the primary sequence level, but share key protein features. *S. cerevisiae* Rif1 consists of 1916 amino acids residues, has a molecular mass of 218 kDa, and contains four identifiable functional domains (**Figure 2**):

**(1) RVxF/SILK protein phosphatase 1 (PP1; Glycogen 7 (Glc7) in budding yeast) docking site 8.** This site contains short KSVAF (residues 114-118) and SILR (146-149) signature sequences 6, which conform to PP1-docking motifs of the RVxF-type (consensus sequence [R/K]x[V/I/]x[F/W], where x denotes any amino acid except large hydrophobic residues) and SILK-type ([G/S]IL[R/K]) 9 (**Figure 2A**). A second putative RVxF/SILK domain has been identified (residues 316-320 and 222-225) 10,11. While the position of the RVxF/SILK domains varies across organisms (**Figure 3**), yeast and mammalian Rif1 orthologs bind PP1, delivering phosphatase activity to origins of replication to exert control over origin firing and DNA replication timing (see **Box 1** for details) 10,12,13,14,15,16,17,18. While there is currently no evidence for an involvement of PP1 binding in the function of Rif1 in promoting DSB repair 19, a recent report indicates that Rif1-dependent recruitment of Glc7 has a role in controlling telomere homeostasis 11.

**BOX 1:**
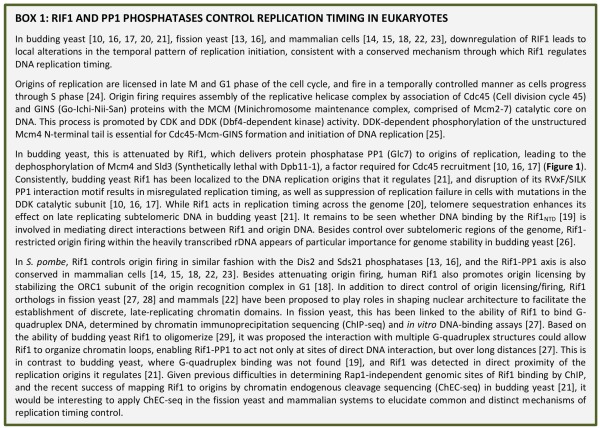
BOX 1: RIF1 AND PP1 PHOSPHATASES CONTROL REPLICATION TIMING IN EUKARYOTES

**(2) The Rif1 N-terminal domain (NTD).** In budding yeast, this domain is preceded by the RVxF/SILK motif and starts after residue 150 (**Figure 2A**), whereas the NTD starts at the very N-terminus of RIF1 in vertebrates 6. The crystal structure of Rif1_NTD_ has recently been solved (residues 177-1283), showing that this domain assumes an elongated fold composed of 23 irregular α-helical repeat units containing a mixture of two-helix HEAT (Huntingtin, elongation factor 3, protein phosphatase 2A, Tor1)-like and three-helix armadillo-like modules 19. Overall, Rif1_NTD_ resembles the shape of a shepherd’s crook, with the hook formed by the N-terminal end (residues 185-874; referred to as Rif1_HOOK_) (**Figure 2B**). Rif1_HOOK_ is the most evolutionary conserved part of Rif1 and corresponds to Pfam domain Rif1_N (PF12231; residues 241-649) 30 (**Figure 3**). A high-affinity DNA-binding site in budding yeast Rif1 was identified within the highly positively charged concave face of the HOOK domain. Rif1_NTD_ was co-crystallized with DNA, showing that Rif1_NTD_ assembles into a head-to-tail dimer such that each HOOK domain forms a DNA binding channel in the resulting figure-8-shaped conformation, and this arrangement was confirmed in solution (**Figure 2C**). Rif1_NTD_ binds DNA in a sequence-independent manner, and associates with double-stranded (ds) and single-stranded (ss) DNA with nanomolar affinity. Direct DNA binding was the first activity that could be ascribed to the NTD and has been linked to a range of Rif1 genome maintenance functions, including telomere homeostasis and DNA repair 19 (explained in more detail below). Recently, murine RIF1 N-terminal DNA binding has been reported 31.

**Figure 4:**
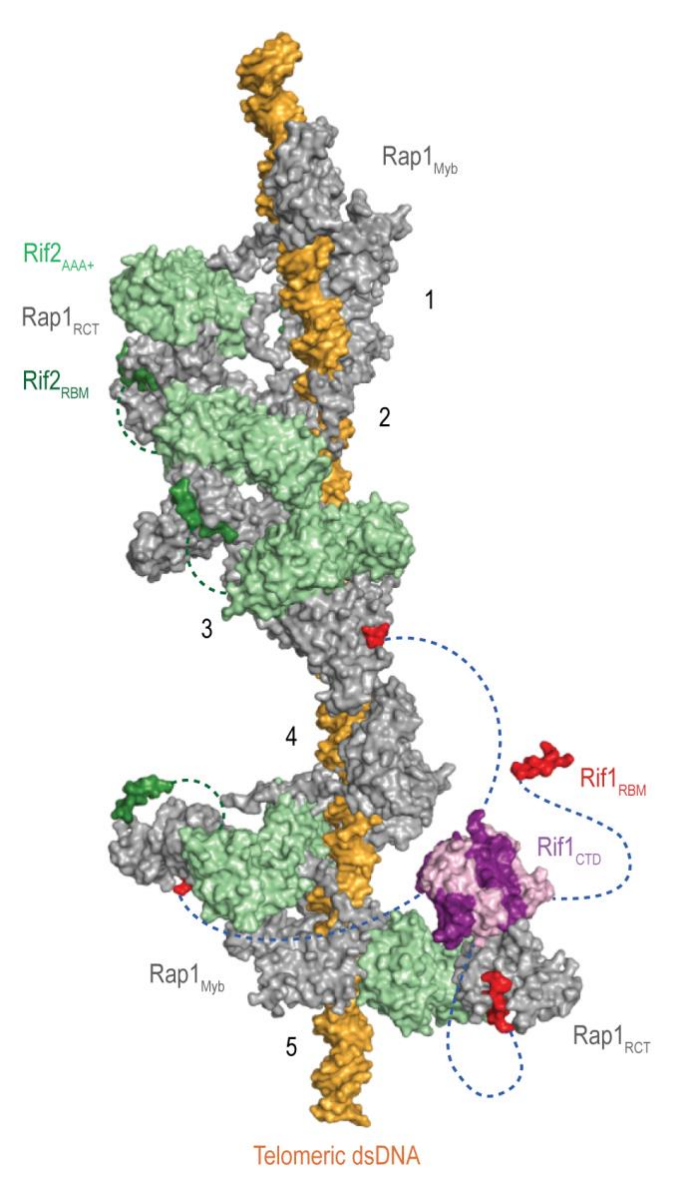
Figure 4: The Velcro-like protein network found at *S. cerevisiae* telomeres. Structural model illustrating the Rap1-Rif1-Rif2 interactions at dsDNA regions of budding yeast telomeres 29. Rap1 (*grey*) engages DNA through its Myb domain (PDB: 1IGN 41). Numbers 1 to 5 indicate Rap1-binding sites. Rif1 and Rif2 are recruited via the Rap1_RCT_ domain (Rap1 C-terminal domain; linker regions not shown; PDB: 4BJ5 29; PDB: 3UKG 42). In this example, each Rap1_RCT_ is bound with Rif2 (PDB: 4BJ1 29) via the Rif2_AAA+_ domain (*light green*). The Rif2_AAA+_ domain is connected to the Rif2_RBM_ (*dark green*), a second Rap1 interaction epitope with similar affinity for Rap1_RCT_ 29. A relatively short linker between Rif2_AAA+_ and Rif2_RBM_ (*green dotted line,* maximal length of 42 Å) limits Rif2 to interlinking neighboring Rap1 molecules. At binding site 5, more complex Rap1-Rif1-Rif2 interactions are shown in exemplary fashion. The Rif1_RBM_ (*red*, PDB: 4BJT) represents the major Rap1 interaction motif; the Rif1_CTD_ (*purple*, PDB: 4BJS) plays an accessory role in Rap1 binding and serves as a tetramerization domain 29. An extended flexible linker (*blue dotted line*) connects the RBM and CTD domains, allowing multimeric Rif1 to interlink up to four Rap1 units over long distances (maximal distance of 110 Å). The Rif1_RBM_ and Rif2_RBM_ bind Rap1_RCT_ in a mutually exclusive manner at the same hydrophobic cleft. In addition, RBM-bound Rap1_RCT_ can engage the Rif1_CTD_ or Rif2_AAA+_ domains. These multipoint interactions between interconnected Rap1, Rif1, and Rif2 stabilize the telosome and have been likened to a molecular Velcro 29. The Rap1_BRCT_ and Rif1_NTD_ domains have been omitted for clarity.

**(3) Rap1 (Repressor/activator site-binding protein 1)-binding motif (RBM, residues 1752-1772).** Rap1 binds the dsDNA TG_1-3_ repeats at budding yeast telomeres in a sequence-specific manner and recruits Rif1 into the telosome (**Figure 4**). This interaction is essential for Rif1 to maintain telomere homeostasis, and disruption of Rif1_RBM_, which constitutes the main Rap1-binding epitope within Rif1, results in telomere dysfunction 29 (explained in more detail below). In fission yeast (*Schizosaccharomyces pombe*), Rif1 is recruited to telomeres by Taz1 (Telomere-associated in *S. pombe* 1) 2, and mammalian RIF1 is not part of the protective shelterin complex at telomeres 34; this can explain why the Rif1_RBM_ motif is only found in *Saccharomycetes*.

**(4) The Rif1 C-terminal domain (CTD).** In budding yeast, crystal structure analysis has shown that Rif1_CTD_ (residues 1857-1916) is a tetramerization module, as well as a secondary Rap1-binding interface 1,29 (**Figure 2A**). In contrast to Rif1_RBM_, the CTD is partially conserved from yeast to human. Specifically, the vertebrate CTD can be subdivided into three regions, CTD-I, II, and III (I: amino acids 2170- 2246, II: 2274-2344, III: 2370-2446; residue numbers refer to human RIF1) (**Figure 3**), and CTD-II corresponds to Rif1_CTD_ in budding yeast. CTD-I contains the canonical mammalian RVxF/SILK motif, while CTD-II was shown to have micromolar DNA-binding activity 31,35,36. Moreover, mammalian RIF1_CTD_ mediates an interaction with Bloom’s syndrome helicase BLM, potentially linking RIF1 to replication stress-induced DNA damage repair 24.

## BUDDING YEAST RIF1 REGULATES TELOMERE HOMEOSTASIS 

### Rif1 underpins telomere architecture

Budding yeast telomeres contain on average 300 base pairs of repetitive TG_1-3_ DNA, which is bound by 15-20 Rap1 molecules. Rap1 contains three domains including a BRCT (BRCA1 C-terminal) domain, the Rap1 C-terminal domain (RCT), and a tandem myb-type helix-turn-helix domain through which the protein engages dsDNA. The short (12-15 nucleotides) telomeric ssDNA TG_1-3_ 3ʹ overhangs are bound by the CST complex, composed of Cdc13 (Cell division cycle 13), Stn1 (Suppressor of cdc thirteen 1), and Ten1 (Telomeric pathways with Stn1). Rap1 and CST are essential genes and hypomorphs of these proteins lead to telomere dysfunction 37.

Rif1 interacts with the Rap1_RCT_ through its RBM and CTD domains. The Rap1-binding epitope RBM is also found in Rif2 (Rap1-interacting factor 2) and the Sir (Silent information regulator) proteins, which are involved in transcriptional silencing 1,29,38,39,40. The Rif1, Rif2, and Sir3 RBMs insert into a hydrophobic cleft within Rap1_RCT_ in a mutually exclusive manner 29. In addition, Rif1 and Rif2 possess secondary Rap1_RCT_-interaction modules: the Rif1_CTD_ (as described above), and a AAA+ (ATPase family associated with diverse cellular activities) domain within Rif2 29. Through its RBM and AAA+ domains, Rif2 can interlink adjacent Rap1 molecules, while Rif1, thanks to an extended linker between its RBM and CTD domains, can engage distant Rap1 proteins. Upon tetramerization, mediated by the CTD, Rif1 can bind up to four Rap1 molecules. Each individual Rap1-binding module within Rif1 and Rif2 is required for telosome stability, indicating the importance of the interconnected, Velcro-like Rap1-Rif1-Rif2 protein network for telomere architecture and function 29 (**Figure 4**).

At native telomeres, Rif1 and Rif2 regulate telomere length by inhibiting telomerase *in cis* 38,43. Similarly, when telomeric DNA sequences are inserted at a chromosome-internal locus that is then cleaved to expose a DNA end flanked by TG_1-3_ repeats, elongation of these telomeric sequences is attenuated by Rif1 and Rif2 44,45. Thus, Rif1 contributes to the regulation of telomerase, an enzyme which adds simple sequence repeats to chromosome ends in order to counteract telomere shortening due to the end-replication problem and nucleolytic degradation 46. It has been observed that telomerase preferentially associates with, and elongates, short telomeres, indicating that long telomeres inhibit telomerase association more efficiently. The "protein counting" model postulates a negative feedback loop, by which the stochastic association of telomerase is increasingly suppressed when increasing amounts of Rap1, Rif1, and Rif2 are present 43,47,48,49,50,51. Conversely, reduced Rap1-Rif1-Rif2 occupancy at short telomeres increases the chance of telomere elongation, while Rap1 phosphorylation at short telomeres provides a mechanism to strengthen Rap1-Rif1 interactions and Rif1 occupancy at telomeres 52. With telomere elongation coinciding with replication, there is also the possibility that the unimpeded progression of replication forks through shorter telomeres may favor telomerase association and telomere elongation 53,54,55,56. Both of these models are compatible with the Velcro-like interactions of Rap1-Rif1-Rif2, which are expected to lead to tighter telomere DNA packaging, and thus resistance to processing and/or replication factors, as telomere length (and with it Rap1-Rif1-Rif2 occupancy at chromosome ends) increases 29.

### Rif1 inhibits telomere elongation by direct DNA binding

While the Velcro-like interactions of the Rap1-Rif1-Rif2 protein network are essential in establishing telomere architecture, the recently discovered Rif1_NTD_ DNA-binding activity proved to be equally important for telomere length regulation 19. It has been demonstrated that disruption of the major Rap1-interaction epitope in Rif1 (the Rif1_RBM_) drastically reduces Rif1 occupancy at telomeres and results in telomere elongation 29. In contrast, Rif1_NTD_ mutations, which reduced Rif1’s ability to bind DNA, affected telomere association less severely, but had a much stronger effect on telomere elongation, phenocopying a *RIF1* deletion 19. These observations revealed a first Rap1-independent role for Rif1 in maintaining telomere homeostasis, showing that while Rap1 interactions are important to assemble Rif1 at chromosome effectively, the ability of Rif1 to bind DNA is crucial to gate access of telomerase.

### Rif1 suppresses checkpoint activation at telomeres

In contrast to DSB ends, where the DDR initiates a network of signaling events that block cell-cycle progression and promote DNA repair 57, telomeric ends are protected from checkpoint activation by their capping complexes. Telomere uncapping in budding yeast by mutations in the CST complex exposes chromosome ends to the DDR, resulting in checkpoint activation 58,59,60,61,62,63. Under these conditions, an attenuation of the DDR by Rif1 can be appreciated. For example, cells carrying the hypomorphic *cdc13-1* allele suffer progressive degradation of the 5ʹ-terminated strand at telomeres 59, but a full-blown DDR is prevented by Rif1, such that cells are saved from terminal checkpoint arrest and survive 64. Rif1’s ability to suppress the lethality associated with Cdc13 dysfunction is dependent on the ability of Rif1_NTD_ to bind DNA, while depletion of Rif1 led to telomere hyperresection 19. Consistently, Rif1 localizes to the ssDNA/dsDNA end-resection junction and reduces the accumulation of ssDNA-binding protein RPA (Replication protein A), dampening recruitment and activation of the apical checkpoint kinase Mec1 (Mitosis entry checkpoint 1; ATR in human) 65. Analogously, Rif1 counteracts the DDR at a model of critically short telomeres, where a DSB is induced at a short, ectopic telomeric DNA repeat sequence 45,66, and can facilitate checkpoint adaptation and cell-cycle re-entry in the presence of DNA damage 67. The anti-checkpoint function of Rif1 in the critically-short telomere model proved to be dependent on the ability of Rif1_NTD_ to bind DNA 19. These observations strongly indicate that direct Rif1-DNA interactions underpin Rif1’s ability to dampen the DDR, potentially by contributing to the assembly of a proper telomere capping architecture and/or competitive exclusion of end-resection factors and checkpoint activators.

### RIF1 and telomeres in mammals

RIF1 does not interact with the telomeric capping complex in mammalian cells, localizing to telomeres only if these are uncapped or critically short 4,5,34. Binding at dysfunctional telomeres likely reflects the involvement of mammalian RIF1 in DSB repair rather than telomere-specific roles.

Nonetheless, there is an interesting link between RIF1 and telomere maintenance in mouse embryonic stem cells. In these cells, RIF1 is highly expressed 3 and helps restrict the expression of the *ZSCAN4* (Zinc-finger and SCAN domain-containing 4) gene. *ZSCAN4* encodes a protein that supports a recombination-dependent telomere-elongation mechanism active in mouse embryonic stem cells 68,69. RIF1 binds to the *ZSCAN4* promoter, where it interacts with components of the methyltransferase complex mediating histone H3 lysine 9 methylation (H3K9me). Thus, RIF1 facilitates H3K9me and a transcriptionally silent chromatin state at the *ZSCAN4* locus, suppressing hyperrecombination, telomere elongation, and chromosome aberrations 69.

## RIF1 IN DNA DOUBLE-STRAND BREAK REPAIR

### Two conserved pathways mediate DSB repair

**Figure 5:**
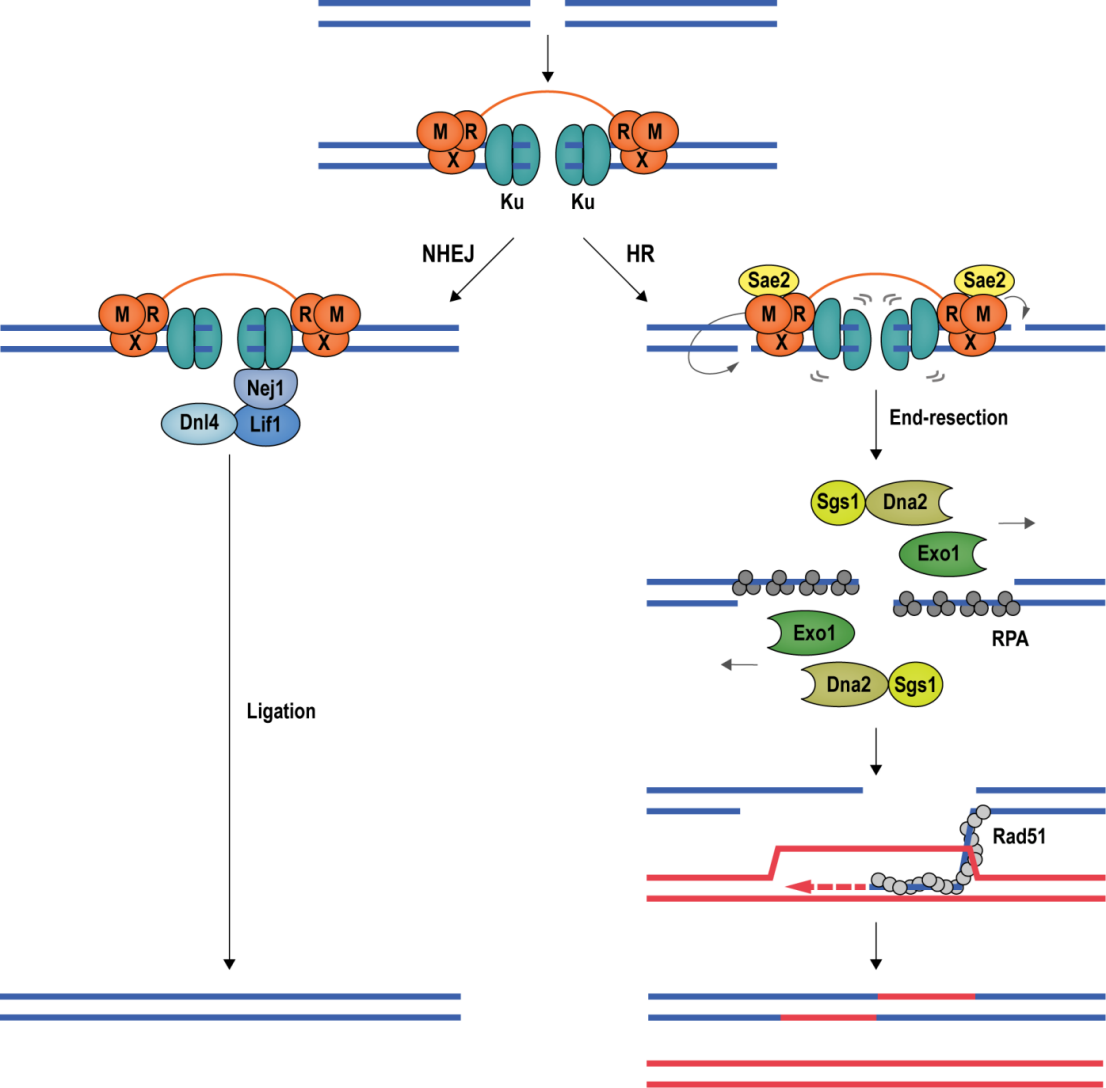
FIGURE 5: DSB repair by NHEJ and HR. The conserved mechanisms of DSB repair by NHEJ and HR are illustrated with budding yeast proteins. *Left*; Ku encircles DSB ends, recruiting the Lif1-Dnl4 and Nej1 ligase complex to promote NHEJ. *Right*; Stimulated by phosphorylated Sae2, Mre11 introduces nicks in the 5ʹ-terminated DNA strands, destabilizing Ku. At the nicks, Exo1 and Sgs1-Dna2 initiate long-range end-resection, exposing ssDNA, which is first coated by RPA, then by recombinase Rad51. The Rad51 nucleoprotein filament seeks out and invades a homologous donor sequence to initiate DNA repair synthesis. HR leads mostly to non-crossover products with a repair patch where new DNA has been synthesized.

DSB repair proceeds via NHEJ or HR 70,71 (**Figure 5**). NHEJ is initiated by the association of the Ku heterodimer (Yku70 and Yku80) with DNA ends. Ku tightly encases DNA ends as a ring-like structure 72, forming a barrier to DNA degradation 73,74. Moreover, Ku serves as a scaffold for the recruitment of the core NHEJ machinery, comprised of the Dnl4-Lif1 (DNA ligase 4 and ligase-interacting factor 1) ligase complex and Nej1 (Non-homologous end-joining defective 1) 75,76,77,78. When the Ku/Dnl4-Lif1/Nej1 complex is stably formed, the break ends are aligned and ligated. NHEJ can occur in all cell-cycle phases and is the preferred repair pathway in G1 and early S phase 70 (**Figure 5**).

DSB repair by HR requires a homologous repair template, which is usually provided by the unbroken sister chromatid 79,80; thus, HR is mainly used for DSB repair during late S and G2 phase of the cell cycle 71. In budding yeast, the first HR factor observed at DSBs is the MRX complex 81, constituted by Mre11 (Meiotic recombination 11), Rad50 (Radiation sensitive 50), and Xrs2 (X-ray sensitive 2) (MRE11, RAD50, and NBS1 (Nijmegen breakage syndrome gene 1) in mammals). Stimulated by phosphorylated Sae2 (Sporulation in the absence of Spo eleven 2; CtIP (CtBP-interacting protein) in mammals) and Ku, Mre11 introduces an endonucleolytic nick on the 5ʹ-terminated DNA strand, initiating DNA end-resection, which leads to the eviction of Ku 82,83,84,85,86,87. Exonuclease Exo1 and the helicase/nuclease complex constituted by Sgs1 (Slow growth suppressor 1; BLM in mammals) and Dna2 (DNA synthesis defective 2) 88 then catalyze long-range 5ʹ to 3ʹ end-resection 89,90. In mammalian cells, MRE11 has been reported to cut both strands of the DNA in close proximity of the break, removing ends occluded by Ku and allowing EXO1 to engage for long-range end-resection 91. The resection tracts are initially covered by RPA, which is then exchanged for the central recombinase Rad51 (Radiation sensitive 51) with the help of recombination mediator proteins. The resulting Rad51 nucleoprotein filament conducts homology search, seeking out a homologous template for DSB repair 92,93 (**Figure 5**).

### DNA end-resection at DSBs determines repair pathway choice

At DSBs, repair pathway choice is intricately linked with DNA end-resection. As described above, NHEJ requires limited, if any, end processing, and becomes inefficient when DNA ends are extensively resected 94. In contrast, HR is dependent on end-resection and exposure of ssDNA tracts, which serve as substrate for the recombination machinery. End-resection is therefore a commitment to DSB repair by HR, and in eukaryotes this commitment is linked to the cell cycle and CDK (Cyclin-dependent kinase) activity 95. In budding yeast, the sole CDK involved in cell-cycle control, Cdc28, regulates end-resection at DSBs 96,97. Cdc28 phosphorylates Sae2 98, which stimulates Mre11 in nicking the 5ʹ-terminated DNA strand, providing an entry site for the end-resection nucleases Exo1 and Dna2 82,84,85. Cdc28 also phosphorylates Dna2, promoting long-range end-resection 99, and the chromatin remodeler Fun30 (Function unknown now 30), which counteracts an end-protection activity exerted by DDR mediator Rad9 100,101. As a result, end-resection and HR-mediated DSB repair are prevalent in late S and G2/M phases of the cell cycle, when CDK activity is high.

Similar CDK-dependent mechanisms promote end-resection in mammalian cells 94. Importantly, phosphorylation of mammalian Sae2 ortholog CtIP promotes its interaction with pro-resection factor BRCA1 (Breast cancer gene 1) 102,103,104. RIF1, in conjunction with 53BP1 (p53-binding protein 1) 105, counterbalances the BRCA1-CtIP axis of end-resection. The role of RIF1 in blocking end-resection and promoting NHEJ in mammalian cells 106 is the subject of the next section.

### An antagonism between 53BP1-RIF1 and BRCA1-CtIP mediates DSB repair pathway choice in mammalian cells

**Figure 6:**
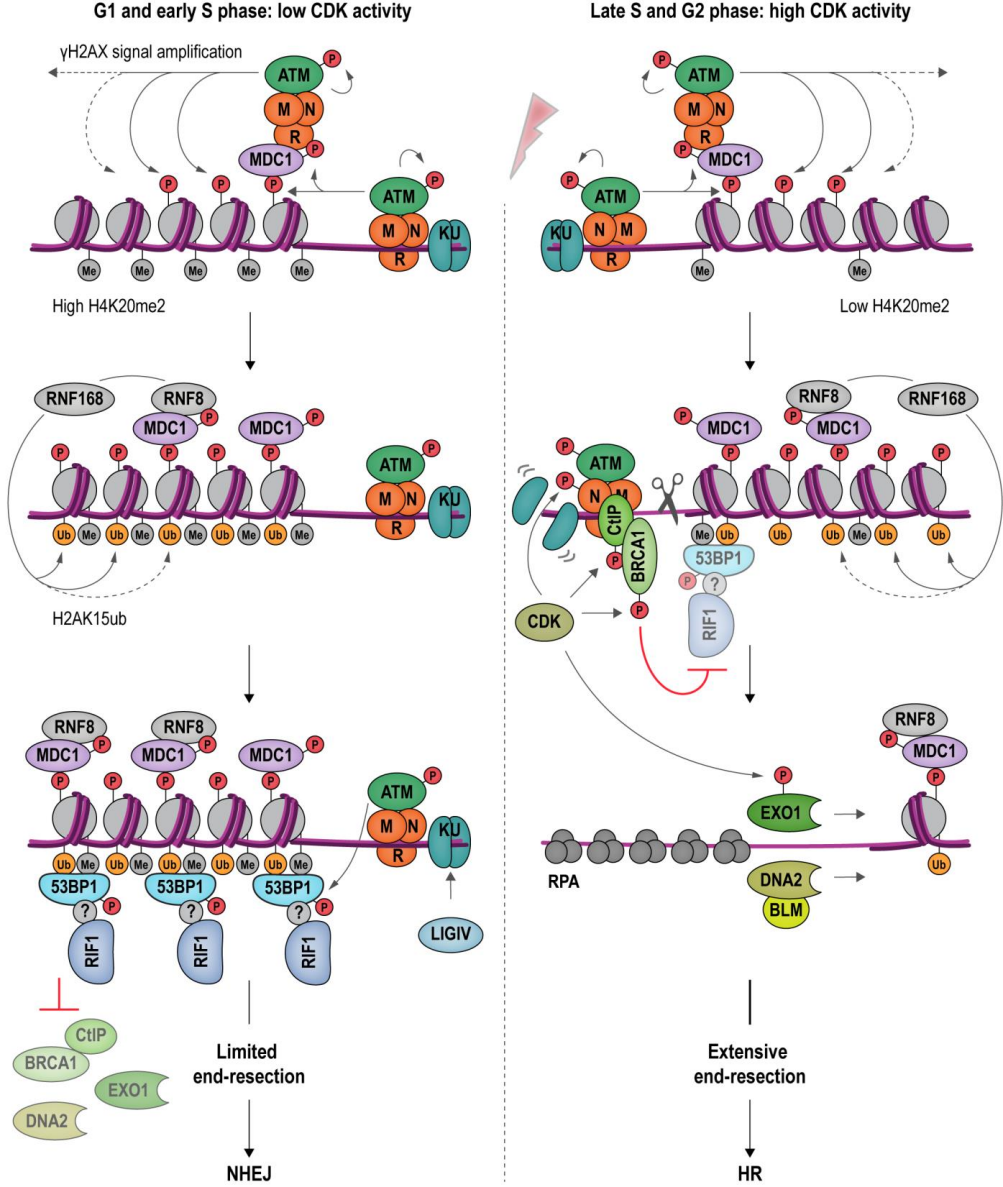
FIGURE 6: An antagonism between 53BP1-RIF1 and BRCA1-CtIP regulates DSB repair pathway choice in mammalian cells. DSB formation triggers a range of protein modifications that orchestrate the cellular response and DNA repair. MRN binds DSBs and recruits apical DDR kinase ATM, which phosphorylates H2AX (γH2AX). γH2AX attracts MDC1 (Mediator of DNA damage checkpoint protein 1), which becomes phosphorylated by ATM and binds additional MRN and ATM, providing a positive feedback loop for signal amplification. MDC1 also recruits RNF8, which cooperates with RNF168 to catalyze protein ubiquitylation at DSBs. H2AK15ub, together with H4K20me2, mediates binding of 53BP1 at DSBs. In its ATM-phosphorylated form, 53BP1 interacts with RIF1, although it remains to be determined whether this interaction is direct. The 53BP1-RIF1 complex blocks resection and inhibits BRCA1-CtIP, EXO1 and DNA2 through an as yet unidentified molecular mechanism. Attenuation of resection results in NHEJ repair in G1 and in early S phase (*left*). In late S and G2 phase, CDK activity rises and the H4K20me2 mark is diluted as a consequence of new histone deposition during DNA replication. CDK stimulates the endonucleolytic activity of the MRN complex, and the recruitment of BRCA1-CtIP to damaged chromatin, while 53BP1-RIF1 binding is diminished. CDK-phosphorylated EXO1 and DNA2-BLM promote long-range resection, generating 3ʹ-ssDNA overhangs, the substrate for the HR-dependent DSB repair machinery (*right*).

RIF1 accumulates at DSBs in a manner dependent on apical DNA damage-checkpoint kinase ATM (Ataxia-telangiectasia mutated) and 53BP1 4, a protein related to checkpoint mediator Rad9 in budding yeast 107 (**Figure 6**). ATM phosphorylates a cluster of 28 N-terminal S/T-Q sites within 53BP1 to promote RIF1 binding 4,108,109,110,111,112,113,114,115,116,117, but whether the interaction between RIF1 and 53BP1 is direct or involves as-yet unidentified accessory factors is currently not clear 118. 53BP1 is a reader of multiple histone marks, allowing 53BP1-RIF1 recruitment to damaged chromatin surrounding DSBs in a highly controlled manner: (1) the tandem Tudor domain of 53BP1 interacts with dimethylated lysine 20 on histone 4 (H4K20me2) 119, and (2) the UDR (ubiquitylation-dependent recruitment) motif contacts mono-ubiquitylated lysine 15 on histone 2A (H2AK15ub) 120,121; (3) it has also been reported that the C-terminal tandem BRCT (BRCA1 C-terminal) domain of 53BP1 interacts with ATM-phosphorylated histone H2AX (γH2AX) 122, however, as the BRCT domain appears to be dispensable for the recruitment of 53BP1 to damaged chromatin 111, the underlying functional relevance of this interaction remains to be elucidated. The H2AK15ub mark is specific to damaged chromatin and deposited by a ubiquitylation cascade involving RING-finger proteins RNF8 and RNF168 (**Figure 6**). H4K20me2 is a constitutive histone mark, which is diluted during replication due to new histone deposition in S phase. 53BP1 recruitment to sites of damage is therefore favored in G1 and less efficient in newly replicated chromatin in late S phase or G2 123,124. Moreover, L3MBTL1 (Lethal 3 malignant brain tumor-like protein 1) 125 and KDM4A (Lysine specific demethylase 4A) 126 compete with 53BP1 for the binding of H4K20me2, while TIRR (Tudor-interacting repair regulatory protein) binds 53BP1, masking the interaction surface for methylated H4 127. Recruitment of 53BP1 to chromatin is further controlled by post-translational modifications deposited on 53BP1 itself. Thus, RNF168-dependent ubiquitylation 128, and PRMT1 (Protein arginine N-methyltransferase 1)-dependent methylation 129 of 53BP1 favors its accumulation at sites of damage, while 53BP1 phosphorylation 130,131 and acetylation 132 reduce the affinity of 53BP1 for H2AK15ub.

Observations showing that downregulation of 53BP1 induces ectopic BRCA1 recruitment to DSBs in G1, and that conversely, depletion of BRCA1 or CtIP leads to accumulation of 53BP1 at chromosomal breaks in G2, indicate that 53BP1 and BRCA1 compete for DSB binding 116. CDK-dependent phosphorylation of CtIP 133,134,135 favors the formation of a complex containing BRCA1, CtIP and MRN 103 at DSBs. BRCA1 and its partner BARD1 (BRCA1-associated RING domain protein 1) form an E3 ubiquitin ligase, adding ubiquitin chains to H2A. Ubiquitylated H2A attracts chromatin remodeler SMARCAD1 (SWI/SNF-related matrix-associated actin-dependent regulator of chromatin subfamily A containing DEAD/H box1; Fun30 in budding yeast), which in turn evicts and repositions nucleosomes and 53BP1 at DSBs 136. Ubiquitylation of RIF1, promoted by BRCA1 interactor UHRF1 (Ubiquitin-like, con-taining PHD and RING finger domains 1), is also involved in dissociating RIF1 from DSB ends 137. Following removal of 53BP1 and RIF1, end-resection, catalyzed by EXO1 138,139 and DNA2-BLM 140,141,142 can take place, initiating HR repair.

While 53BP1 and RIF1 are epistatic in repressing end-resection at DSBs 113,117, it is not yet understood how 53BP1 and RIF1 cooperate to inhibit the end-resection machinery and promote NHEJ mechanistically. By analogy to budding yeast, where Rif1 participates in organizing higher-order structures at telomeres 29 (**Figure 4**), it has been speculated that 53BP1 and RIF1 might arrange DSBs into structures less accessible for resection 118. At uncapped telomeres, resembling one-ended DSBs, RIF1’s role in attenuating end-resection is supported by BLM 35 and MAD2L2 (Mitotic spindle assembly checkpoint protein MAD2B) 143, and these interactions could putatively play similar roles at chromosome breaks. Finally, given the interaction between RIF1 and PP1 (see** Box 1**), it is tempting to speculate that the dephosphorylation of DDR and/or resection factors may be involved in RIF1-dependent attenuation of end-resection 106.

### Rif1 and DSB repair pathway choice in budding yeast: Rif1 attenuates DNA end-resection by tightly encasing DNA ends

The involvement of *S. cerevisiae* Rif1 in DSB repair has emerged only recently. A first indication that budding yeast Rif1 may localize to broken DNA came from studies focused on the telomeric roles of Rif1. In a model for critically short telomeres, where a DSB is generated proximal to a short telomeric repeat sequence, Rif1 accumulates in a Rap1-dependent manner. Surprisingly, Rif1 was observed at these telomeric breaks even when its C-terminal Rap1-interaction modules (the Rif1_RBM_ and Rif1_CTD_ domains) were disrupted, which suggested a Rap1-independent mechanism of recruitment 29. This was confirmed by findings of Rif1 targeting induced DSBs at different places in the budding yeast genome, and in a manner fully independent of telomeric DNA sequences 19,67,144.

In contrast to the mammalian system, a first analysis of budding yeast Rif1 at non-telomeric DSBs showed that cells deleted for *RIF1* accumulated less ssDNA at distances greater than ~2 kb from a break site in G1 phase of the cell cycle 144. This correlated with increased binding of DNA damage checkpoint mediator Rad9, a protein known for its ability to function as a barrier to end-resection 145,146. It therefore appears that Rif1 may facilitate longer-range end-resection by limiting Rad9 recruitment under certain conditions. While this Rif1-Rad9 interplay has been shown to be important for deleterious intrachromosomal break repair, any impact on canonical DSB repair by HR or NHEJ remains unclear 144,147.

Further insight into the interaction of Rif1 with DSBs resulted from the identification of the Rif1_NTD_ DNA-binding site (**Figure 2**). *In vitro*, Rif1_NTD_ binds dsDNA and ssDNA substrates in a sequence-independent manner, showing preference for 3ʹ-tailed ssDNA-dsDNA junctions, a DNA structure similar to those found at telomeric ends and DSB ends. As mentioned above, direct DNA binding by the Rif1_NTD_ was found to play critical *in vivo* roles by counteracting telomerase and the attenuation of end-resection at uncapped telomeres in budding yeast. Analogously, and mirroring the situation in mammalian systems, Rif1_NTD_ also engages DSBs, attenuates end-resection, and promotes NHEJ 19. In yeast strains harboring an inducible DSB that can only be repaired by NHEJ 148,149, loss of Rif1 destabilized the break ends and reduced repair by ~40%. End-protection and the promotion of NHEJ by Rif1 was dependent on the DNA-binding activity residing in the HOOK domain of Rif1_NTD_; in contrast, the Rap1 and PP1-binding modules are apparently not required 19. These findings show that the role of Rif1 in modulating DSB repair pathway choice is evolutionary conserved, and that the yeast and mammalian Rif1 orthologs are functionally more similar than previously thought.

The structural and functional evidence in budding yeast strongly suggests that Rif1_NTD_ promotes NHEJ by tightly encasing DNA ends in a way that sterically excludes the end-resection machinery. In human, the NTD is strictly required for recruitment of Rif1 to DSBs, while the C-terminal part of the protein contributes moderately 116. Based on the evolutionary conservation of the Rif1_NTD_ 6 and recent reports of DNA binding by the murine RIF1 31, it is tempting to speculate that Rif1 may operate in DSB repair by gating access to DNA ends across organisms.

### Possible means of regulation of budding yeast Rif1 in DSB repair

In mammalian cells, the actions of RIF1 in DSB repair pathway choice are dependent upon 53BP1 113,115,116,117. A potential functional equivalent to 53BP1 in budding yeast is Rad9. Like 53BP1, Rad9 is a reader of histone marks, interacting with damaged chromatin through its Tudor and BRCT domains binding H3K79me 150,151 and γH2AX 152,153, respectively. So far, no functional or physical interactions between Rad9 and Rif1 analogous to the mammalian 53BP1-RIF1 axis have been reported. Quite to the contrary, Rif1 has been shown to prevent the accumulation of Rad9 at telomeres, inhibiting the DDR 65,66. As described above, a similar antagonism between Rif1 and Rad9 may operate at DSBs 67,144. How yeast Rif1 is regulated in NHEJ is an interesting open question. HEAT repeats have frequently been linked with protein-protein interactions 154, raising the possibility that, in addition to direct DNA binding, Rif_NTD_ may mediate as-yet unknown physical interactions regulating its functions in NHEJ.

In budding yeast, Rif1 has been seen in foci at the nuclear periphery, coinciding with the subnuclear position of telomeres 155,156,157. Fission yeast Rif1 has been proposed to establish late-replicating domains by dictating specific chromatin architectures in proximity of the inner nuclear membrane 27 (see **Box 1**). Similarly, mouse RIF1 proved critical in linking nuclear spatial organization and replication timing, and based on the observation that RIF1 interacts with Lamin B1, this function may be exerted by physical interactions with the inner nuclear membrane 22. Given that nuclear compartments are important for DSB repair 158,159, it will be interesting to explore whether a nuclear-peripheral localization of Rif1 may be functionally involved in NHEJ.

## CONCLUSIONS AND FUTURE PERSPECTIVES

**Figure 7:**
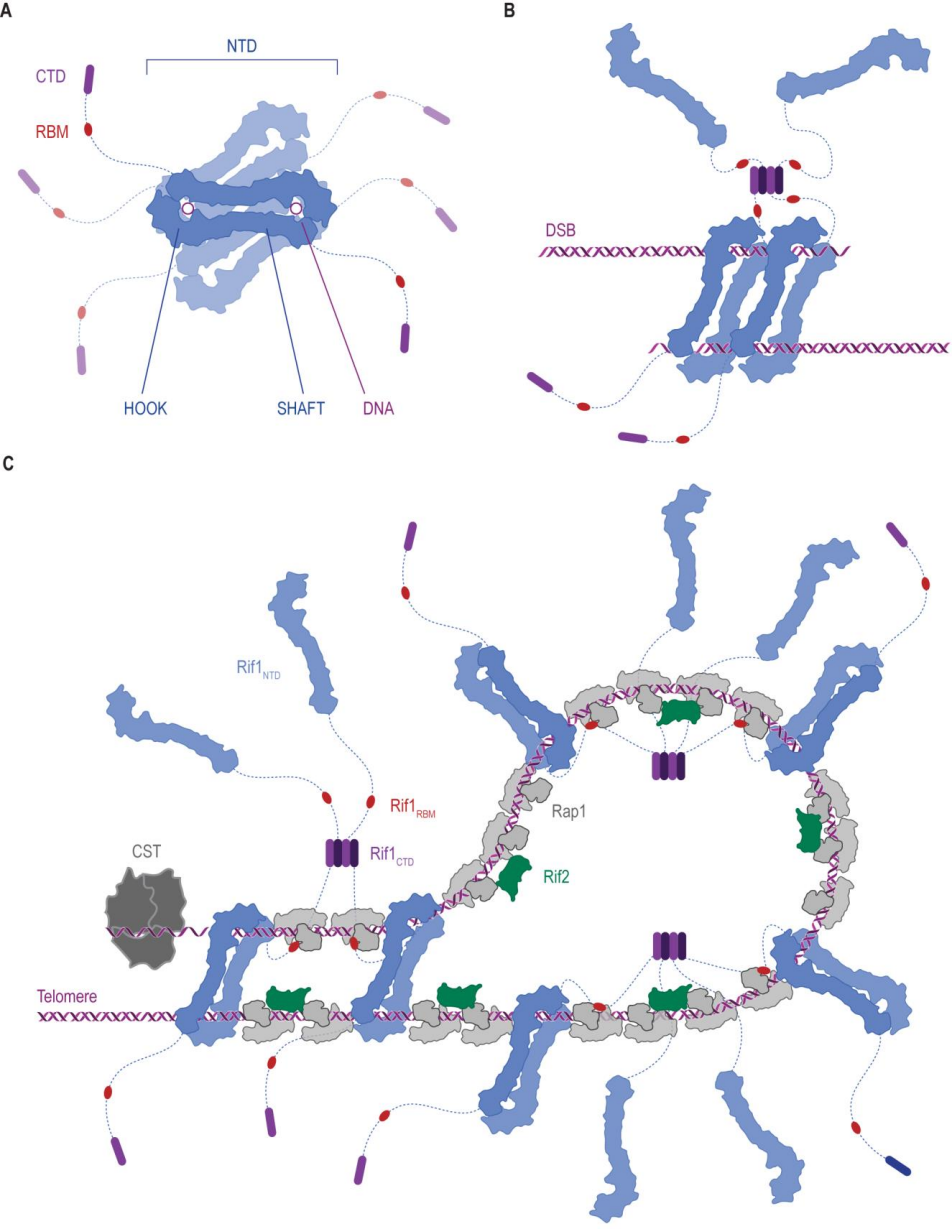
FIGURE 7: Ways in which Rif1 dimerization may promote DSB repair and shape telomere architecture. **(A)** Crystallographic model showing budding yeast Rif1 dimers bound to two DNA molecules 19. The shepherd’s crook-like Rif1_NTD _(*blue*) comprises an N-terminal HOOK and a straight SHAFT region. Rif1_RBM_ (Rap1-binding domain,* red*) and Rif1_CTD_ (C-terminal tetramerization domain,* purple*) are connected to Rif1_NTD_ by unstructured linker regions (*dotted lines*). Rif1_NTD_ has intrinsic DNA-binding activity and assembles on DNA as a figure 8-shaped, head-to-tail dimer. Multiple dimers may be organized around the same DNA molecules, forming a protein sheath, and restricting access of other proteins. **(B)** Speculative model of Rif1 dimers binding to the two ends of a DSB: tethering DSB ends in this way may promote re-ligation along the NHEJ pathway by keeping DSB ends in close proximity. **(C)** Schematic representation of the Velcro-like protein network formed by Rap1, Rif1 and Rif2 at yeast telomeres, taking DNA binding by Rif1 into account. Rap1 molecules (*grey*) directly bind dsDNA TG_1-3_ tracts, recruiting Rif1 and Rif2. Rif2 (*green*) interlinks adjacent Rap1 molecules, while Rif1, forming tetramers via its CTD, engages multiple Rap1 molecules through RBM epitopes. The NTD may allow Rif1 to directly engage telomeric DNA at sites not covered by Rap1. Fold-back structures could potentially be stabilized by Rif1-mediated DNA-bridging.

Initially described as a telomeric protein in budding yeast 1, Rif1 is now recognized as a key genome maintenance factor that exists across eukaryotes. A growing body of evidence indicates that most of Rif1’s diverse functions are evolutionary conserved. Studies in yeast 10,13,16,17,20,21 and mammalian cells 14,15,18,22,23 have provided a detailed view of Rif1 in the regulation of replication timing by shared mechanisms involving control over origin firing and chromatin structure (**Box 1**). Pioneering work in mammalian cells has established RIF1 as a mediator of DSB repair pathway choice 4,5,113,115,116,117. At DSBs, RIF1 promotes NHEJ by attenuating end-resection, although the mechanism of action in mammalian cells remains to be elucidated. The yeast model may prove informative in these efforts. Budding yeast Rif1 has long been regarded purely as a telomere maintenance factor, tethered to chromosome ends by DNA-binding protein Rap1 1,29. With recent studies linking budding yeast Rif1 to DSB repair, this view has changed 19,67,144. Detailed structural analyses have revealed direct DNA binding by the conserved NTD of Rif1, which, in budding yeast at least, mediates functionally important interactions with chromosome ends and DSB ends alike 19. The crook-shaped Rif1_NTD_ encases DNA ends (**Figure 2 **and **Figure 7A**), gating access of processing factors. This provides an elegant, direct mechanism that allows Rif1 to control diverse biological processes including telomere elongation and end-resection. Rif1_NTD_-mediated DNA binding may be conserved in mammals 31, and it will be important to establish how generally the mechanistic insights from budding yeast apply to other eukaryotes.

Rif1 binds DNA in oligomeric form 19,31. In the Rif1-DNA co-crystal, Rif1_NTD_ is seen in a head-to-tail dimer configuration bound with two DNA molecules 19 (**Figure 2C **and** Figure 7A**). The significance of this intriguing arrangement remains to be elucidated *in vivo*, but it is tempting to speculate that multipoint DNA interactions may underpin Rif1 function. For example, tethering DSB ends, as seen in case of the MRX 160,161,162,163,164,165,166 and MRN complexes 167,168,169,170, could be involved in Rif1’s role in promoting NHEJ (**Figure 7B**). At telomeres, higher-order chromatin structure is important for homeostasis. In mammalian cells, chromosome ends fold back on themselves and display a lariat-like structure (T-loops), generated by invasion of the 3ʹ ssDNA overhang into the dsDNA region of the telomere 171. In human, shelterin component TRF2 (Telomeric repeat-binding factor 2) is crucial in forming and maintaining T-loops 171,172. Similar fold-back structures exist in yeast, and in budding yeast Rif1 and Rif2 are implicated in their formation 173,174,175,176,177. Although speculative at the moment, both Rif1 multimerization 29 and multi-point DNA binding 19, could promote the stability of higher-order telomere structures (**Figure 7C**), and by analogy may also support higher-order RIF1-53BP1 assemblies at repair sites.

In conclusion, Rif1 is emerging as a versatile and multifaceted genome maintenance protein involved in DNA replication timing, telomere maintenance, and the repair of chromosome breaks. It now appears that its cellular functions are largely conserved among eukaryotes. The way in which Rif1 is integrated into the telomere protective cap is unique to yeast. Yet, the finding that Rif1 utilizes an intrinsic DNA-binding activity within the conserved Rif1_NTD_ to regulate telomere length and end-resection at DSBs is compatible with the view that a conserved DNA repair protein has been "hijacked" and is moonlighting in telomere homeostasis 178. The mechanistic parallels of direct DNA binding by Rif1 in DNA repair and telomere maintenance provide a satisfyingly unified view of Rif1 shepherding DNA ends to safeguard genome stability.
